# Multiple steps of leaf thickening during sun‐leaf formation in Arabidopsis

**DOI:** 10.1111/tpj.14467

**Published:** 2019-09-09

**Authors:** Rina Hoshino, Yuki Yoshida, Hirokazu Tsukaya

**Affiliations:** ^1^ Department of Biological Sciences Graduate School of Science The University of Tokyo Bunkyo‐ku Tokyo 113‐0033 Japan; ^2^ Exploratory Research Center on Life and Living Systems National Institutes of Natural Sciences Okazaki Aichi 444‐8787 Japan

**Keywords:** cell morphology, cryptochromes, developmental plasticity, light response, phototropins, ploidy, sugar response

## Abstract

Plant morphological and physiological traits exhibit plasticity in response to light intensity. Leaf thickness is enhanced under high light (HL) conditions compared with low light (LL) conditions through increases in both cell number and size in the dorsoventral direction; however, the regulation of such phenotypic plasticity in leaf thickness (namely, sun‐ or shade‐leaf formation) during the developmental process remains largely unclear. By modifying observation techniques for tiny leaf primordia in *Arabidopsis thaliana*, we analysed sun‐ and shade‐leaf development in a time‐course manner and found that the process of leaf thickening can be divided into early and late phases. In the early phase, anisotropic cell elongation and periclinal cell division on the adaxial side of mesophyll tissue occurred under the HL conditions used, which resulted in the dorsoventral growth of sun leaves. Anisotropic cell elongation in the palisade tissue is triggered by blue‐light irradiation. We discovered that anisotropic cell elongation processes before or after periclinal cell division were differentially regulated independent of or dependent upon signalling through blue‐light receptors. In contrast, during the late phase, isotropic cell expansion associated with the endocycle, which determined the final leaf thickness, occurred irrespective of the light conditions. Sucrose production was high under HL conditions, and we found that sucrose promoted isotropic cell expansion and the endocycle even under LL conditions. Our analyses based on this method of time‐course observation addressed the developmental framework of sun‐ and shade‐leaf formation.

## Significance Statement

Thick sun leaves or thin shade leaves develop in response to light intensity, but the mechanism involved in leaf‐thickness growth is unclear. Based on high‐resolution sectional images of leaf primordia, we identified two distinct developmental stages. During the early stage, intense light triggers changes in cell shape via blue‐light receptors. During the later stage, sucrose enhances the isotropic enlargement of cells. Taken together, these results provide insight into sun‐leaf formation.

## Introduction

Light levels directly affect plant morphology and physiology. Plants can adapt to a wide range of light conditions by changing their photosynthetic apparatus (Gauhl, 1976; Boardman, 1977; Yano and Terashima, 2001). According to the light intensity, the leaf internal structure also exhibits changes in the adaxial–abaxial or dorsoventral direction (Coulter, 1900; Talbert and Holch, 1957). In ecological and anatomical terms, thick leaves induced under high light (HL) conditions are called ‘sun leaves’, whereas thin leaves formed under low light (LL) conditions are called ‘shade leaves’ (Haberlandt, 1914). These leaf types are thought to be determined by unknown systemic light signals transduced from mature leaves to developing leaves: i.e. mature leaves sense the surrounding light intensity and convert light information to systemic signals that determine the leaf type of developing primordia (Uemura *et al*., 2000; Yano and Terashima, 2001). This hypothesis has been supported by irradiation with different light intensities of the shoot apex and mature leaves of several species, indicating that common or similar mechanisms underlie this regulation (Uemura *et al*., 2000; Yano and Terashima, 2001; Munekage *et al*., 2014). Such regulation is important because adjusting the thickness of the leaf modifies the proportion of photosynthetic tissue per leaf area for the efficient absorption of light energy (Boardman, 1977; Terashima *et al*., 2005).

The thickening of sun leaves is characterized by several typical morphologies of the leaf internal structure. For example, the tree fern *Cyathea caracasana* forms thick sun leaves by increasing the number of cell layers in mesophyll tissue (Arens, 1997). In the sun leaves of *Solanum dulcamara*, anisotropic cell elongation in the palisade tissue contributes to leaf thickening (Gauhl, 1976). A combination of an increase in the number of cell layers and in the anisotropic elongation of mesophyll cells is observed in the sun leaves of *Arabidopsis thaliana* (hereafter Arabidopsis) in the Brassicaceae and *Chenopodium album* in the Amaranthaceae (Weston *et al*., 2000; Yano and Terashima, 2001). Although the anatomy, ecology, and physiology of sun and shade leaves have been studied in many plant species, the mechanism by which plants control leaf thickening remains unclear from a developmental perspective (Tsuge et al.,^1996^; Donnelly et al., 1999; Yano and Terashima, 2004; Kalve et al., 2014; Chitwood et al., 2015).

In plant organogenesis, cell number is regulated by mitotic cycle activity, whereas cell size is thought to be determined by cell growth (an increase in cytoplasmic volume) during the mitotic phase and cell expansion (an increase in vacuole volume) in the post‐mitotic phase, often associated with the endoreduplication cycle in Arabidopsis (Traas *et al*., 1998; Kondorosi *et al*., 2000; Sugimoto‐Shirasu and Roberts, 2003; Tsukaya, 2008). The endoreduplication cycle or endocycle, a modified cell cycle causing repeated DNA replication without nuclear division, is affected by light quantity and quality in plant organogenesis (Gendreau *et al*., 1998; Komaki and Sugimoto, 2012; Scholes and Paige, 2015; Okello *et al*., 2016). In Arabidopsis hypocotyls, anisotropic cell elongation is associated with endocycle progression in the dark, which is in turn repressed under light through the functions of phytochrome and cryptochrome photoreceptors (Gendreau *et al*., 1998); however, the endocycle is not always the driving force underlying cell size and shape determination in plant organogenesis (Tsukaya, 2008). For example, several rounds of endocycle constantly occur regardless of cell size control in leaf petioles (Kozuka *et al*., 2005). The size of leaf mesophyll cells is mostly uniform, whereas the variation of their ploidy levels is comparable with that of epidermal cells, the size of which shows marked variation (Katagiri *et al*., 2016). These findings suggest that the number of endocycles is not simply proportional to the cell size in leaves.

In sun leaves of Arabidopsis, cells in the palisade mesophyll are larger than those in shade leaves through anisotropic elongation in the dorsoventral direction under HL conditions (Wuyts *et al*., 2012). Also, HL irradiation of LL‐grown seedlings promotes the endocycle (Mohammed *et al*., 2018), but whether endocycle progression is responsible for anisotropic cell elongation in sun leaves is unknown. This is because of the scarcity of basic information on leaf‐thickness growth, in contrast to that gleaned from extensive studies on paradermal growth in leaves (Tsuge *et al*., 1996; Donnelly *et al*., 1999; Yano and Terashima, 2004; Tsukaya, 2013; Kalve *et al*., 2014; Chitwood *et al*., 2015).

To investigate the formation of sun and shade leaves during plant development, we focused on the spatiotemporal aspects of leaf thickening. We devised a suitable method for observing the internal structure of tiny leaf primordia and used this method to trace the process of leaf thickening through time‐course observations. We also assessed the roles of candidate factors involved in leaf‐thickness growth.

## Results

### Structure and ploidy levels of mature sun and shade leaves

**Figure 1:**
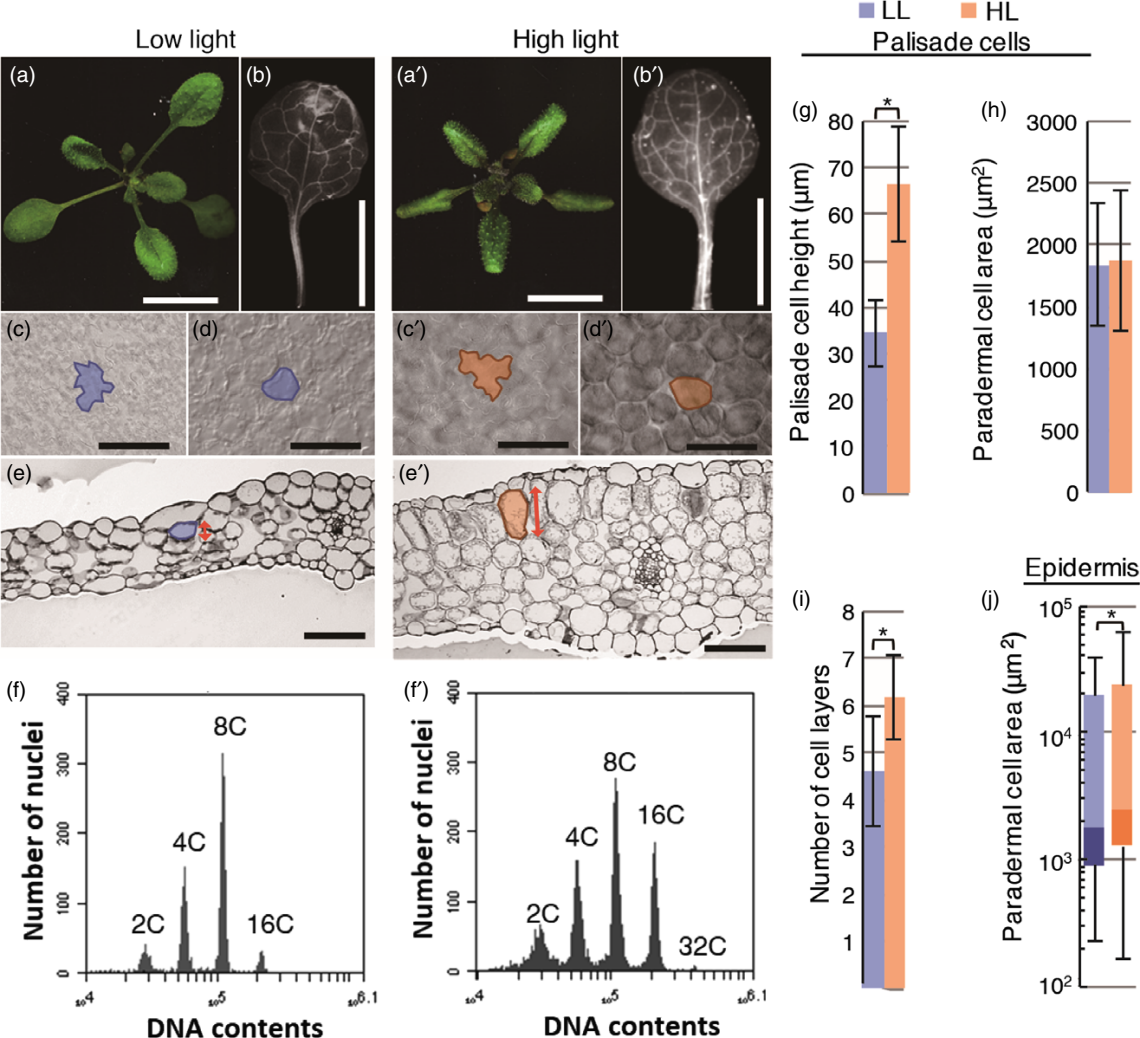
Leaf morphology and ploidy levels of mature sun and shade leaves in wild‐type Arabidopsis. Shade (a–f) and sun leaves (a′–f′) were induced under low and high light conditions for 21 days after sowing (DAS). (a, a′) Rosette morphology. (b–f, b–f′) Matured leaf and cell shape of the first pair of foliage leaves. Paradermal image of adaxial epidermal cells (c, c′) and first layer of palisade cells (d, d′). A typical cell of each leaf type is outlined. Cell area of each cell type obtained from the paradermal images was quantified in (h) and (j). (j) The top and bottom ends of the lines represent the highest and lowest values observed. Upper, middle and bottom of boxes represent 75%, 50% and 25% values of each plot. Quantifications of palisade cell height (g) and number of cell layers in the leaf thickness direction (i) obtained from leaf transverse section (e, e′). (g) Cell height is cell length in dorsoventral direction indicated as a double‐headed arrow in (e) and (e′). Values represent means ± SDs (*n* = 10 leaves from five plants). Asterisks indicate statistical differences by Student's *t*‐test (*P *<* *0.0001). (f, f′) Ploidy profiles of the first foliage leaf at 21 DAS. In the ploidy measurement, each sample contained only a single leaf blade. The above result had been replicated in at least three independent experiments. Scale bars: (a–b, a′–b′) 1 cm; (c–e, c′–e′) 100 μm.

To induce sun and shade leaves, here we set LL at 70 μmol photons m^−2^ s^−1^ and HL at 280 μmol photons m^−2^ s^−1^. In sun leaves induced under HL conditions, multiple layers of palisade cells were anisotropically elongated along the adaxial–abaxial axis or in the dorsoventral direction of leaves (Figure 1a′,b′,e′), whereas shade leaves induced under LL conditions had a single layer of palisade tissue composed of small round cells (Figure 1a,b,e). For detailed morphological characterization, we quantified cell size in the palisade and epidermal tissues of fully matured leaves (Figure 1g–j). In the palisade tissue, the cell height (cell length in the dorsoventral direction in cross section, arrows in Figure 1e,e′) of sun leaves was almost twice that of shade leaves (Figure 1e,e′,g), whereas there were no significant differences in cell area in the paradermal plane between the two leaf types (Figure 1d,d′,h). The number of cell layers in the dorsoventral direction was also significantly higher in sun leaves than in shade leaves (Figure 1i). Thus, through a combination of increased cell size and cell number, sun leaves developed remarkably thicker palisade tissue than shade leaves in Arabidopsis. Because these morphological features are common for sun‐ and shade‐leaf differentiation in many plant species (Talbert and Holch, 1957), we focused on these phenotypes as characteristic hallmarks of sun leaves in subsequent experiments. In addition to changes in mesophyll cells, we found that the cell area of epidermal pavement cells differed significantly between sun and shade leaves (Figure 1c,c′,j).

Next, we performed flow cytometry on fully matured sun and shade leaves. Sun leaves had higher ploidy levels than shade leaves (Figure 1f,f′). Whereas shade leaves had four distinct peaks (2C, 4C, 8C, and a small peak at 16C), sun leaves had an additional small peak at 32C (Figure 1f,f′). The mean percentage of 16C cells in sun leaves (34.1 ± 6.0%) was markedly higher than that in shade leaves (9.3 ± 2.3%), with a decrease in the proportion of 8C cells. Thus, HL enhanced the third and fourth round of the endocycle in the leaves. To determine whether large mesophyll cells in sun leaves exhibit specific enhancement of the endocycle, we also measured the ploidy levels of epidermal and mesophyll cells of the same leaf samples by separating the *pATML1::H2B‐mGFP‐*positive and ‐negative fractions (Roeder *et al*., 2010; Katagiri *et al*., 2016). In fully mature leaf samples, there were no clear differences in ploidy levels among epidermal tissue, mesophyll tissue and whole leaf samples grown under the same light conditions (Figure S1a). Therefore, in sun leaves, both the ploidy levels and the cell size of the entire leaf tissue increased compared with those in shade leaves.

### Temporal analysis of leaf thickening during sun‐ and shade‐leaf formation

**Figure 2:**
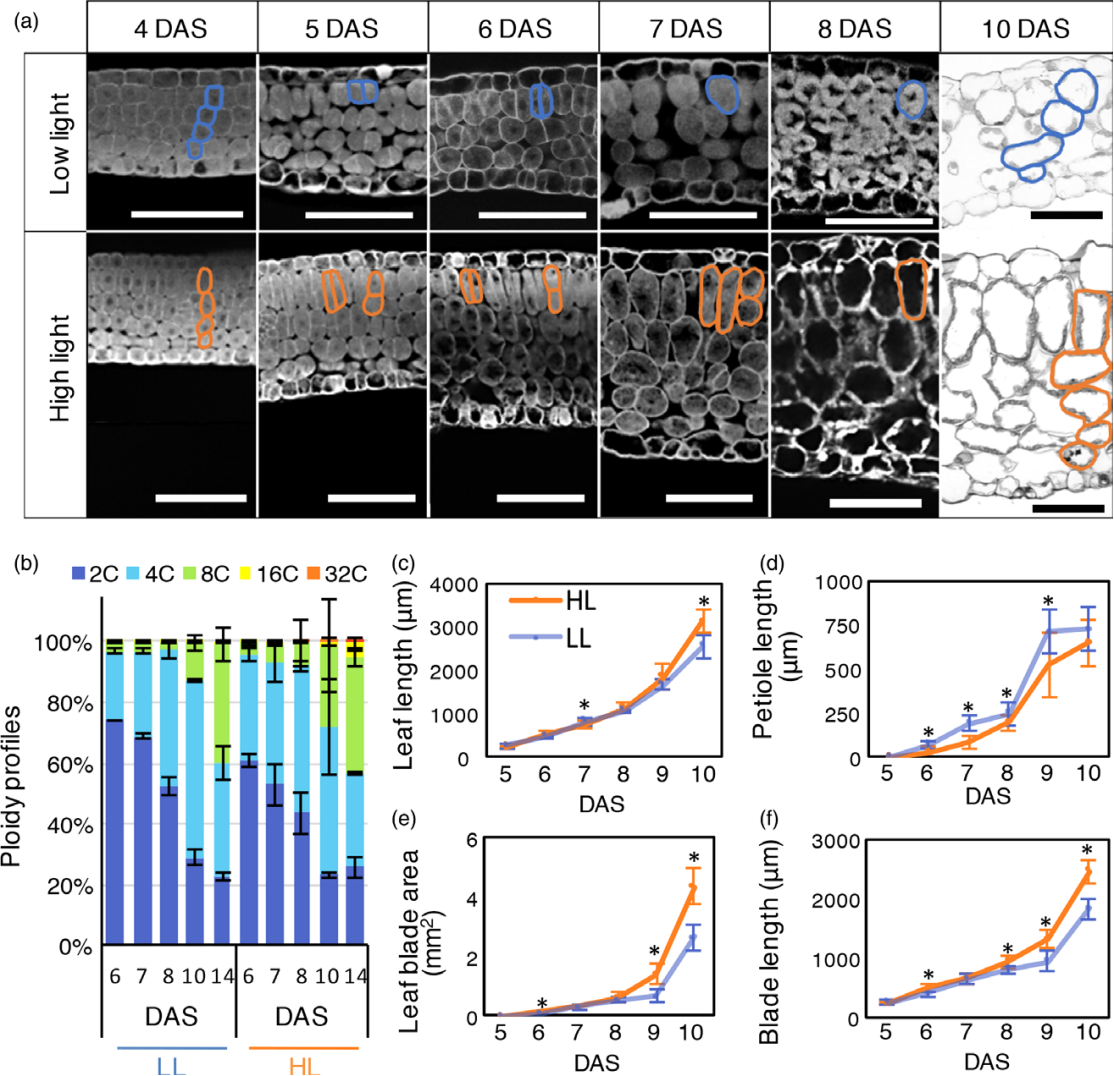
Time‐course analysis of developing sun and shade leaves in the wild type. (a) Longitudinal sections of developing leaf blades in shade leaves (upper: low light) and sun leaves (lower: high light) sampled at 4, 5, 6, 7, 8 and 10 days after sowing (DAS). For morphological observations, images of optical sections were taken under a confocal laser microscope (4–8 DAS) or of Technovit sections under a light microscope (10 DAS). Typical mesophyll tissue cells are outlined at each stage, and changes in cell layers of leaf primordia at 4 and 10 DAS are shown. We analysed three samples per condition, and the most representative data are shown. (b) Ploidy levels of developing sun and shade leaves at 6, 7, 8, 10 and 14 DAS. Samples contained more than 10 first foliage leaves (from five individual plants) at 6–8 DAS or six first foliage leaves (from three plants) at 10–14 DAS. (c) Leaf length, (d) leaf petiole length, (e) leaf blade area and (f) leaf blade length in developing leaves at 5–10 DAS. Values represent means ± SDs (*n* = 10 leaves from five plants). Asterisks indicate statistically significant differences by Student's *t*‐test (*P *<* *0.0001). Scale bars: 50 μm.

The manner and timing of leaf‐thickness growth in sun‐ and shade‐leaf formation have not previously been elucidated owing to the technical difficulties of observing tiny leaf primordia using conventional sectioning methods. To overcome this problem, we modified the sample preparation step for observing leaf thickness using the combination of a tissue‐clearing technique and semi‐whole‐mount observation (details are described in the Experimental procedures and in Figure S2). Using this contrivance, we obtained a series of high‐resolution images of leaf longitudinal sections (Figure S2a–c). To investigate the developmental process of sun and shade leaves, we observed both the outer and internal structures of leaf primordia in the paradermal and dorsoventral directions. Because the first pair of foliage leaves emerges soon after germination (Ichihashi *et al*., 2011), we analysed these leaves from 4 to 8 days after sowing (4–8 DAS). Morphological differences in leaf paradermal growth between LL and HL leaves were detected at 6 DAS (Figure 2c–f). The leaf blades expanded to a larger size under HL than under LL conditions; conversely, leaf petioles were longer under LL than under HL conditions. No differences in leaf‐thickness growth were observed between LL and HL conditions at 4 DAS, but significant differences emerged from 5 DAS: cells in the adaxial subepidermal tissue began to divide and elongate in the dorsoventral direction under HL but not under LL conditions (Figure 2a). Subsequently, from 6 to 7 DAS, both periclinal and anticlinal cell divisions were more frequently observed than at 5 DAS under HL conditions, whereas only anticlinal cell division was observed under LL conditions (Figure 2a). During this period, under HL conditions, the cell heights of palisade cells increased further. Through this anisotropic growth, leaf primordia thickness became greater at 7 DAS under HL than under LL conditions (mean leaf thickness = 101 μm under HL and 68 μm under LL conditions). From 8 to 21 DAS, under both light conditions, cell division ceased, and most mesophyll cells began isotropic expansion with an increase in intercellular space (Figures 1e,e′ and 2a). Through increasing isotropic expansion and intercellular space, the thickness of fully mature leaves doubled during this process (mean leaf thickness under HL = 123 μm at 8 DAS and 249 μm at 21 DAS). In summary, the process of leaf dorsoventral growth can be categorized as follows. Before 5 DAS, leaves remain undifferentiated and show a common internal structure, with four layers of mesophyll cells, irrespective of the light conditions. We defined this pre‐differentiation step as the ‘initiation phase’ (as discussed later in Figure 7). From 5 to 7 DAS under HL conditions, the adaxial mesophyll cells exhibit anisotropic cell elongation in the leaf dorsoventral direction and periclinal cell division. After 8 DAS, adaxial and abaxial mesophyll tissues reach their final thickness through isotropic cell expansion and increased intercellular space. Hereafter, we define 5–7 DAS as the ‘early phase’ and 8 DAS onwards as the ‘late phase’ of leaf‐thickness growth, which differs between LL and HL conditions. The early phase was specifically observed in sun‐leaf formation under HL conditions, whereas the late phase was observed in both shade‐ and sun‐leaf formation under LL and HL conditions, respectively (Figure 7).

We hypothesized that if anisotropic cell elongation in the palisade cells is associated with endocycle progression, ploidy levels should already differ during the early phase of sun‐leaf formation at 5–7 DAS. We measured the ploidy levels of young leaf primordia starting at 6 DAS, as 4‐ to 5‐DAS leaf primordia were too small to perform flow cytometric analysis. From 6 to 8 DAS, the ploidy levels of primordia under HL conditions were only slightly shifted towards higher ploidy levels compared with the ploidy levels under LL conditions (Figure 2b), and these ploidy levels were mainly composed of 2C–4C cells. Because cell division continued to be active at this phase (Figure 2a), G2‐phase cells in the mitotic cycle could contribute to the 4C cells, in addition to endoreduplicated cells. In contrast, the manner in which ploidy changed from 10 to 14 DAS differed between samples under different light conditions (Figure 2b). Under LL conditions at 10 DAS, the second round of the endocycle (namely 4C to 8C) progressed slowly, whereas rapid progression from the second to the third round of the endocycle was seen under HL conditions. From these ploidy analyses, we found that the endocycle progressions were as follows: only a slight difference in the frequency of the first round of the endocycle between LL and HL conditions at 6–8 DAS, and an apparent increase in the second and third rounds of the endocycle under HL compared with LL conditions at 10–14 DAS. We also checked the ploidy levels of mesophyll and epidermal cells separately and confirmed that the ploidy profile of the whole leaf (Figure 2b) was similar to that of the mesophyll samples, irrespective of the light conditions (Figure S1b).

Our time‐course observations and ploidy analyses revealed that changes in cell shape and increases in ploidy levels were triggered at different times during sun‐leaf formation. Anisotropic cell elongation of mesophyll cells occurred from 5 to 7 DAS, whereas major increases in ploidy levels were seen after 10 DAS. Hence, it is unlikely that ploidy contributes to anisotropic cell elongation during sun‐leaf formation in Arabidopsis.

### Sun‐leaf formation in a TOPO VI mutant

**Figure 3:**
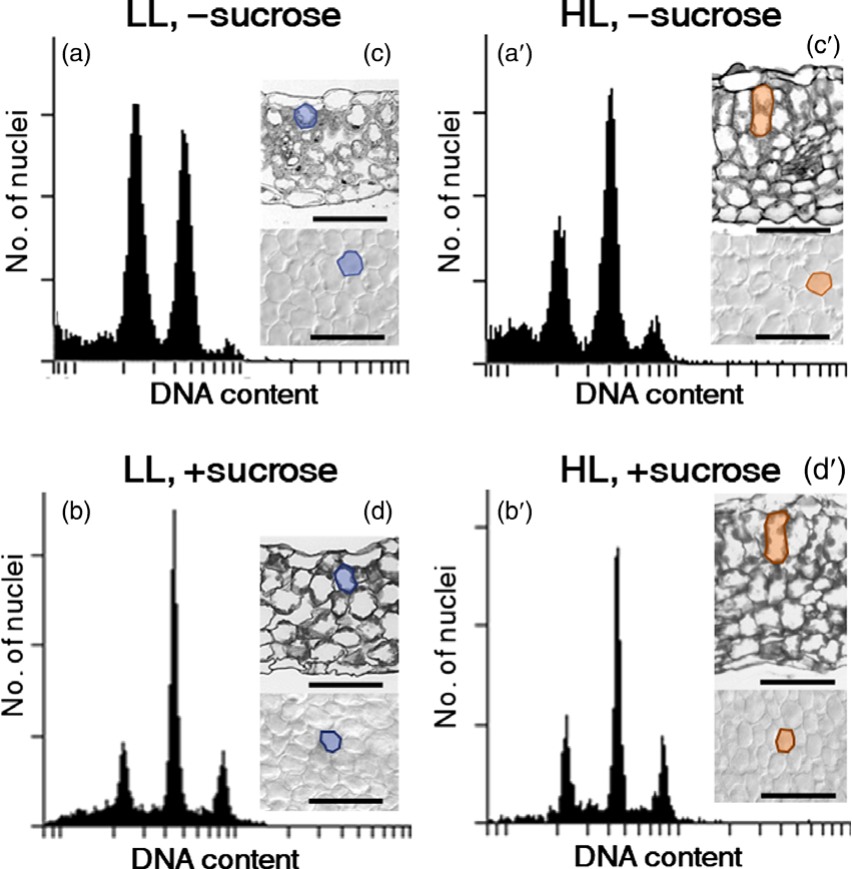
Leaf morphology and ploidy levels of sun and shade leaves in the *bin4* mutant. *bin4* mutant plants were grown under low light (LL) conditions (a–d) and high light (HL) conditions (a′–d′) for 21 days after sowing (DAS) on either rockwool (a, c, a′, c′) or MS plates with 2% sucrose (+sucrose; b, d, b′, d′). (a, a′, b, b′) Ploidy profiles of leaf blades grown under LL (a, b) and HL (a′, b′) conditions. Samples included more than 10 foliage leaves. (c, c′, d, d′) Transverse sections (upper) and paradermal view (lower) of the fourth foliage leaves. Typical palisade cells are outlined at each stage. Representative data from more than three replicates are shown. Scale bars: 50 μm.

To further clarify the role of endocycle progression in sun‐leaf formation under HL conditions, we used the *brassinosteroid‐insensitive 4* (*bin4*) mutant. The *bin4* mutant has a defect in the DNA‐binding subunit of topoisomerase VI and shows severe growth phenotypes associated with strong repression of endocycle progression in leaves and other organs (Yin *et al*., 2002; Breuer *et al*., 2007). We cultivated the *bin4* mutant plants under HL or LL conditions and analysed their leaf structure and ploidy levels (Figure 3a,a′,c,c′). Irrespective of the light conditions, *bin4* plants formed extremely small leaves and even the largest foliage leaf blade was around 1 mm in length (Figure S3; Breuer *et al*., 2007). This decrease in leaf area originated from a decrease in cell size; the paradermal cell area of *bin4* leaves was considerably smaller than that of wild‐type leaves and was unaffected by light conditions (Figures 1a–e,a′–e′ and 3c,c′; Breuer *et al*., 2007). In cross section, however, anisotropic elongation and multiple cell layer formation in the palisade tissue was observed in the *bin4* mutant under HL but not under LL conditions (Figure 3c, c′). Meanwhile, the endocycle was strongly repressed in *bin4*, even under HL conditions; the proportions of polyploid cells above 8C in *bin4* plants (Figure 3a,a′; 5.0% under LL and 9.6% under HL) were comparable with the polyploidy levels of leaf primordia at 8 DAS in the wild type under the corresponding light conditions (Figure 2b). This suggests that *bin4* plants have a defect in the late phase of sun‐leaf formation (endocycle and isotropic expansion), but not in the early phase (anisotropic growth) of leaf thickening.

### Leaf‐thickness growth and ploidy levels in blue‐light receptor mutants

**Figure 4:**
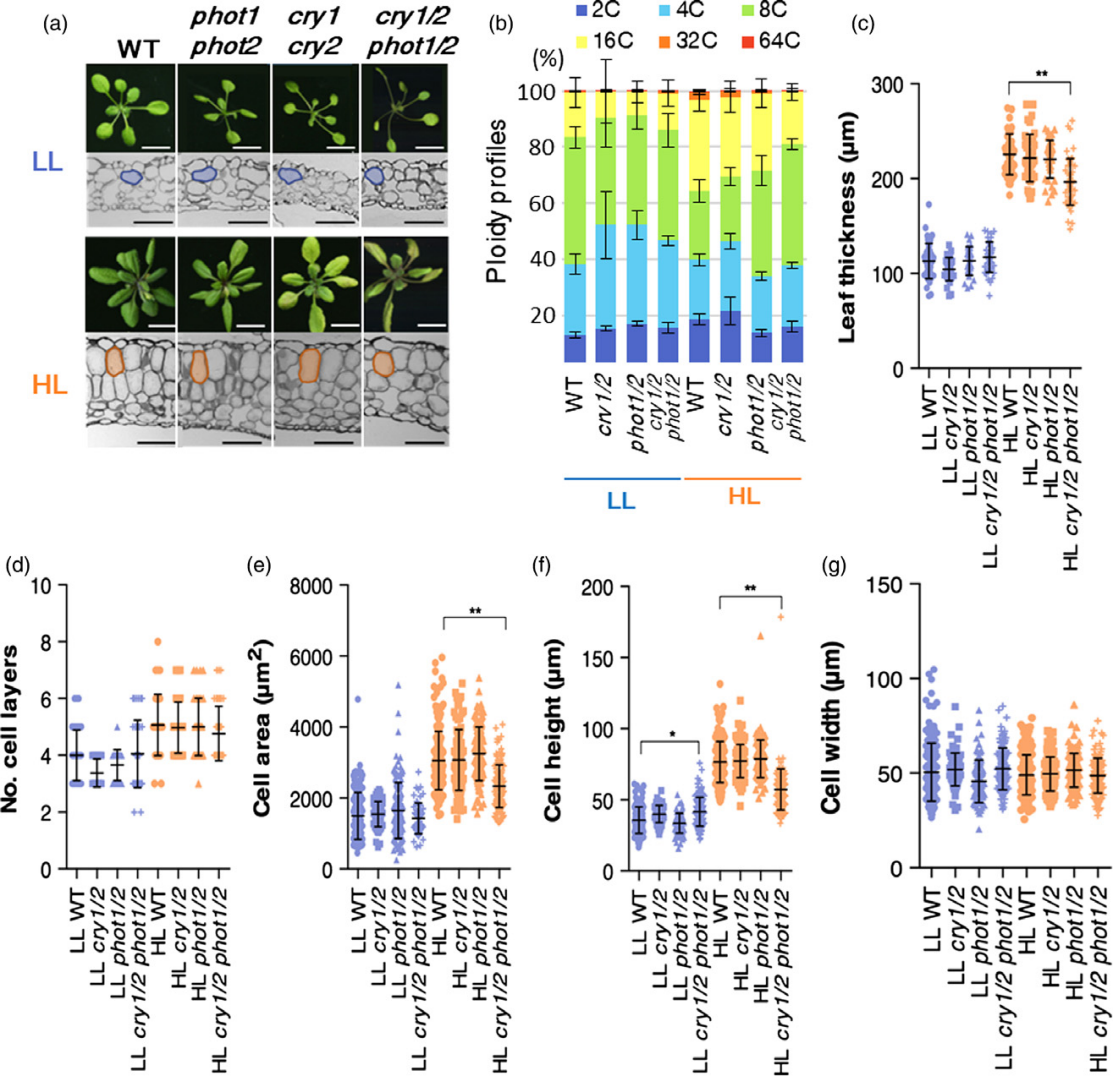
Leaf morphology and ploidy levels of blue‐light receptor mutants under low light (LL) or high light (HL) conditions. (a, b) Wild‐type (WT, *gl1*) and mutant plants with defects in blue‐light sensing, *phot1 phot2* double mutant (*phot1/2*), *cry1 cry2* double mutant (*cry1/2*), and *cry1 cry2 phot1 phot2* quadruple mutant (*cry1/2 phot1/2*), were cultivated under LL and HL conditions for 21 days. (a) Rosette morphology (upper panels) and leaf cross sections (lower panels) of the first pair of foliage leaves in each genotype. Typical mesophyll tissue cells are outlined at each stage. (b) Ploidy profiles of leaf blades of shade and sun leaves of WT and mutant plants. Values represent means ± SDs (*n* = three plants). (c) Leaf thickness, (d) cell layer number, (e) palisade cell area in leaf transverse section, and (f) cell height, and (g) cell width in first layer of palisade cells. Values represent means ± SDs (*n* = 4 or 5 plants). Asterisks indicate statistically significant differences by Sidak's multiple comparison test (*P *<* *0.0001**, 0.001 < *P *<* *0.01*). Scale bars: white bars, 1 cm; black bars, 100 μm.

To elucidate the signalling pathway linking light intensity and anisotropic cell growth during sun‐leaf formation, we next focused on blue‐light receptors. According to a previous study, irradiation with strong red light causes leaf thickening through isotropic cell expansion instead of anisotropic cell elongation, whereas irradiation with strong blue light was sufficient to induce normal sun‐leaf formation, similar to the situation under the white HL conditions used (López‐Juez *et al*., 2007). A class of blue‐light receptors, phototropins, was assumed to be involved in the determination of cell shape by increasing cell height and limiting cell width along the leaf paradermal plane in a tissue‐autonomous manner (Kozuka *et al*., 2011; Munekage *et al*., 2014). Whether the *phot1 phot2* double mutant shows defects in sun‐leaf formation remains under debate, however (López‐Juez *et al*., 2007; Kozuka *et al*., 2011). López‐Juez *et al*. (2007) and Kozuka *et al*. (2011) used *phot1‐101* *phot2‐5* and *phot1‐5 phot2‐1*, respectively. *phot1‐5 phot2‐1* showed defects in anisotropic growth of the palisade tissue (Kozuka *et al*., 2011). Here, we examined sun‐leaf formation in double mutants with the null alleles *phot1‐5 phot2‐1* and *phot1‐5 phot2‐2* (Figure 4a and Figure S4; Kinoshita *et al*., 2001; Kozuka *et al*., 2011; Suetsugu *et al*., 2013). Under our HL conditions, *phot1 phot2* plants formed thick leaves with elongated palisade cells, like wild‐type plants (Figures 4a and S4). Similarly, the *cry1 cry2* double mutant, which lacked another class of blue‐light receptors, also exhibited normal sun‐leaf formation under HL conditions (Figure 4a). To further inhibit blue‐light sensing, we crossed *phot1 phot2* with *cry1 cry2* to construct the *cry1 cry2 phot1 phot2* quadruple mutant. Under HL conditions, *cry1 cry2 phot1 phot2* plants had leaves that were thinner than the leaves of the wild‐type plants (Figure 4a). Also, only the *cry1 cry2 phot1 phot2* mutant showed defects in anisotropic cell elongation under HL conditions (Figure 4e,f), whereas the number of cell layers was comparable with that of the wild type and the double mutants (Figure 4d). Thus, the decrease in leaf thickening in the quadruple mutant was caused by the altered anisotropic cell elongation (Figure 4c–g).

**Figure 5:**
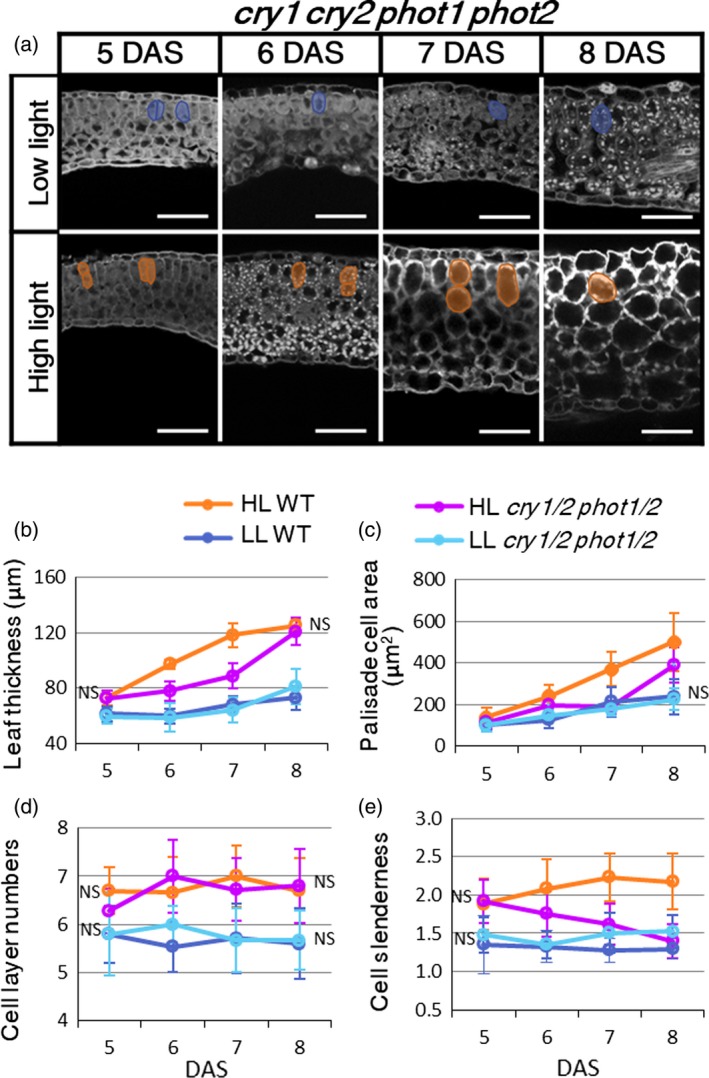
Time‐course analysis of leaf‐thickness growth in *cry1 cry2 phot1 phot2* mutant. (a) Leaf longitudinal sections of *cry1 cry2 phot1 phot2* (*cry1/2 phot1/2*) mutants grown under low light (LL) or high light (HL) conditions from 5–8 days after sowing (DAS). Typical mesophyll tissue cells of each leaf sample are outlined. Scale bars: 50 μm. (b) Leaf thickness, (c) cross‐sectional area of palisade cells, and (d) the number of cell layers were measured from the longitudinal images. (e) Cell slenderness (ratio of cell height and width) in palisade cells was also calculated from the same images. Values represent means ± SDs (*n* > 3 plants). Absence of NS means significant difference by Sidak's test (*P* < 0.05), when the *cry1 cry2 phot1 phot2* is compared with the wild type under each light condition.

To further characterize the *cry1 cry2 phot1 phot2* quadruple mutant, we performed time‐course observations of leaf‐thickness growth (Figure 5a–e). At 5 DAS, the palisade cells of the *cry1 cry2 phot1 phot2* quadruple mutant showed anisotropic elongation under HL conditions, similar to the wild type (Figures 2a and 5a); however, after periclinal cell division in the palisade tissue from 6 DAS, which halves the cell height, cells in the quadruple mutant were round in shape under HL conditions (Figure 5a). During the development of young leaf primordia, leaf thickness and cell layer number showed no significant difference between the quadruple mutant and the wild type (Figure 5b,f). Cell slenderness (cell height‐to‐width ratio) also showed no significant difference between the quadruple mutant and the wild type at 5 DAS; however, that of the quadruple mutant after 5 DAS became significantly smaller under HL conditions only (Figure 5c–e). The transverse cell area also showed a significant difference under HL conditions only (Figure 5c). These data suggest that there are two types of anisotropic cell elongation, before and after 5 DAS, where the latter is lost in the *cry1 cry2 phot1 phot2* quadruple mutant.

We also wondered whether the palisade cell phenotype of the *cry1 cry2 phot1 phot2* mutant was derived from defects in canonical blue‐light signalling pathways or other unknown mechanisms related to photoreceptor function (Galvão and Fankhauser, 2015; Huché‐Thélier *et al*., 2016; Casal and Qüesta, 2017). To investigate this, we tested the effect of monochromatic light irradiation on leaf thickening in blue‐light receptor mutants (Figure S5). A previous study reported that irradiation with monochromatic blue light at 200 μmol photons m^−2^ s^−1^ was sufficient to induce leaf‐thickening growth (López‐Juez *et al*., 2007). We found that under monochromatic light of this intensity, leaves were thinner compared with leaves grown under the white HL conditions as a result of reductions in cell size and the number of cell layers (Figures 4a,c–f and S5a–c). Nevertheless, blue light induced the formation of cylindrical cells whereas red light induced the formation of spherical cells in the palisade tissue, as described previously (Figure S5a,e; López‐Juez *et al*., 2007; Kozuka *et al*., 2011). In *phot1 phot2*, the palisade cell area in leaf transverse sections showed no significant difference, with a decrease in cell height accompanied by a small increase in cell width (Figure S5d,f,g). In *phot1 phot2*, the palisade cell area in leaf transverse sections showed no significant difference, with a decrease in cell height instead of an increase in cell width (Figure S5d,f,g). In the quadruple mutant, both the cell height and the cell width were strongly and significantly reduced under the blue‐light conditions. Under the red‐light conditions, the internal structure of leaves in the examined mutants were identical to that of the wild type; the exception was the *phot1 phot2* double mutant, which exhibited greater isotropic cell expansion (Figure S5a,d,f,g). These results indicate that palisade cell size and shape in sun leaves are controlled through canonical blue‐light signalling mediated by cryptochromes and phototropins.

We also measured the ploidy levels of blue‐light receptor mutants. Under LL conditions, all of the mutants had similar ploidy levels as wild‐type shade leaves (Figure 4b). Under HL conditions, the ploidy levels were slightly lower than those in wild‐type sun leaves in both the *phot1 phot2* and the *cry1 cry2* double mutants, whereas the ploidy levels of the *cry1 cry2 phot1 phot2* quadruple mutant were lower under HL conditions (Figure 4b). Thus, alterations in anisotropic cell elongation and ploidy levels under HL conditions were impaired only when four blue‐light receptors were lost.

### Effects of exogenous sucrose treatment on leaf structure and ploidy levels

The molecular mechanism regulating the number of cell layers during sun‐ and shade‐leaf formation is unclear. According to a previous study, the number of cell layers of the first foliage leaves is determined by the surrounding light conditions experienced before 3 DAS, when seedlings are just unfolding their cotyledons (Kalve *et al*., 2014), suggesting that the number of cell layers is controlled systemically rather than in an organ‐autonomous manner (Yano and Terashima, 2001). We focused on sucrose, a major photo‐assimilated sugar, which is transported from mature leaves (source) to developing organs (sink) and is known to accelerate cell proliferation (Martin *et al*., 1993; Terashima *et al*., 2005). Sucrose stimulates the cell cycle via D‐type cyclins and target of rapamycin1 (TOR1) kinase, which are activated in both the mitotic cycle and the endocycle (Khamlichi *et al*., 2000; Menges *et al*., 2006; Dewitte *et al*., 2007; Velappan *et al*., 2017). Additionally, sucrose is highly synthesized in mature leaves under HL conditions in Arabidopsis and has been proposed as a candidate systemic signal for sun‐leaf induction (Terashima *et al*., 2005; Jänkänpää *et al*., 2012; Mohammed *et al*., 2018).

**Figure 6:**
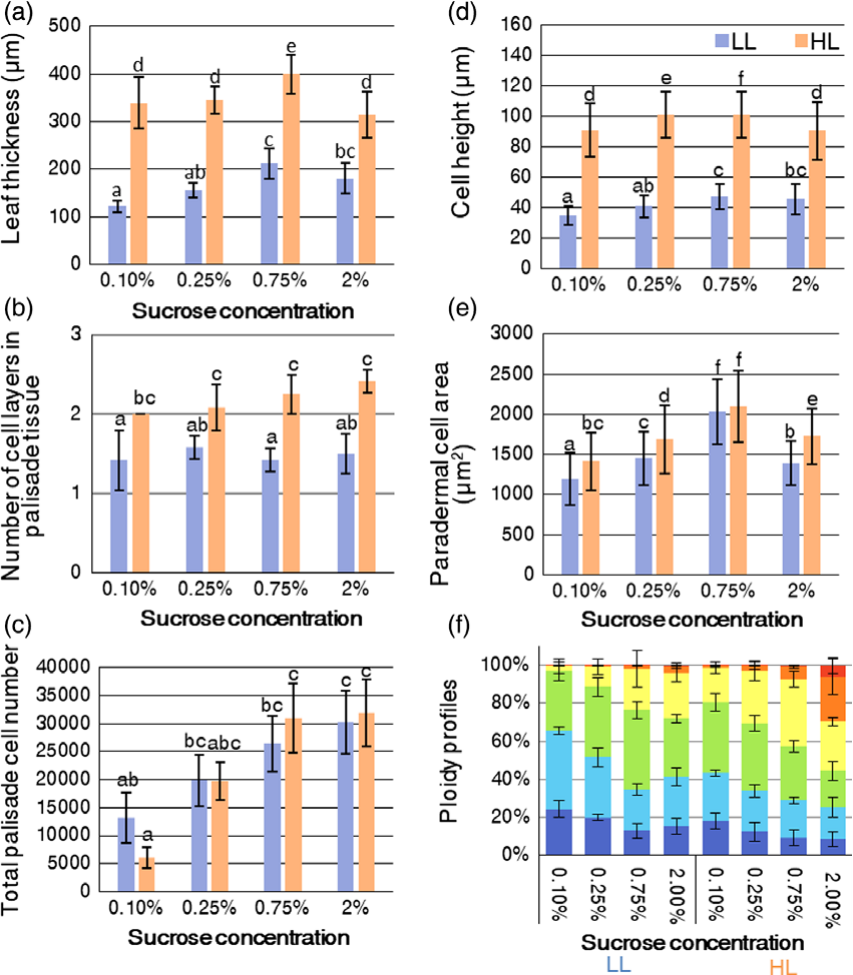
Effects of exogenous sucrose on leaf morphology and ploidy levels in the wild type. The wild‐type seedlings were cultured on MS plates supplemented with a series of sucrose concentrations (0.1–2.0% w/v) under low light (LL) and high light (HL) conditions. The first pairs of foliage leaves were collected at 21 days after sowing (DAS) to quantify anatomical parameters and ploidy profiles. (a) Leaf thickness, (b) the number of cell layers in palisade tissue, (c) total number of cells in palisade tissue per leaf, (d) cell height of palisade tissue, and (e) palisade cell area counted from transverse sections. Significant differences (*P *<* *0.05) are indicated by different letters (Tukey's test). (f) Ploidy profiles. The percentages indicate the sucrose concentrations in each medium. Values represent means ± SDs (*n* = 6 leaves from three plants).

To test the effects of sucrose on leaf thickening, wild‐type seedlings were cultured on MS media with a series of sucrose concentrations (0.1, 0.25, 0.75, and 2% w/v) under HL and LL conditions. We measured several morphological parameters and performed statistical analyses by two‐way analysis of variance (anova) and Tukey's *post hoc* tests to compare the independent effects of light conditions (see Experimental procedures ; Figure 6a–e; Tables S1 and S2). Irrespective of the light conditions, leaf thickness, palisade cell height, and palisade cell area in the paradermal direction were greater in the presence of 0.75% sucrose, compared with 2.00% sucrose, in the growth medium (Figure 6a,d,e). Regarding the effect of osmotic stress, we confirmed that a high concentration of sugar reduced the cell size (Figure S7). The paradermal growth of leaves was promoted by sucrose in a concentration‐dependent manner (Figure 6, S6 and S7; Tables S1 and S2). This effect was caused by the combination of increased paradermal cell area and cell numbers in a sucrose concentration‐dependent manner, whereas these parameters were less affected by light intensity (Figures 6c,e and S7; Tables S1 and S2). Because the total cell number in the palisade tissue per leaf significantly increased in a sucrose concentration‐dependent manner (Figure 6c; Table S2), we concluded that sucrose increases the cell division frequency. Nevertheless, leaf thickness significantly increased in a sucrose concentration‐dependent manner under both LL and HL conditions (Figures 6a and S6). This was attributable to the weak increase in cell height by isotropic expansion in response to sucrose treatment (Figures 6d and S6). In contrast, the number of cell layers in the palisade tissue was predominantly controlled by the light conditions and was unaffected by the sucrose concentration (Figures 6b and S6).

Ploidy levels also increased in a sucrose concentration‐dependent manner, even under LL conditions (Figure 6f). As shown in Figure 2, the endocycle was enhanced when isotropic cell expansion was observed in the late phase of leaf‐thickening growth, suggesting that sucrose may be involved in this late phase. Notably, although the highest ploidy levels were observed when seedlings were treated with 2% sucrose, cell size (both height and paradermal area) decreased under these conditions, regardless of light intensity (Figures 6d,f and S6).

To further examine the effects of sucrose on endocycle activation, *bin4* mutant plants were treated with sucrose (Figures 3b,d,b′,d′ and S3). Without an exogenous supply of sucrose, the ploidy levels of *bin4* plants were lower under LL than under HL conditions (Figure 3c,c′). With sucrose treatment, the ploidy levels of *bin4* plants increased even under LL conditions and reached the same ploidy levels as HL‐grown samples (Figure 3b,b′). Despite the promotional effects of sucrose on endocycle progression, only the first and second rounds of the endocycle were upregulated in the *bin4* mutant. Although BIN4 is required for high levels of endoreduplication, sucrose‐induced endocycle activation was still retained in a BIN4‐independent manner.

## Discussion

### Importance of developmental observations in tracing leaf‐thickening growth

The plasticity of leaf thickness in response to light intensity was described more than a century ago (Coulter, 1900), and this phenomenon has been reported in various plant species (Haberlandt, 1914; Talbert and Holch, 1957). Yano and Terashima (2004) carried out detailed characterizations of sun‐ and shade‐leaf development in *C. album*, but further molecular approaches to identify the causes underlying such differentiation were difficult to achieve with the materials available. Weston *et al*. (2000) and López‐Juez *et al*. (2007) analysed the anatomical and photosynthetic traits of sun leaves in wild‐type Arabidopsis and several mutant lines; however, these studies using Arabidopsis did not fully clarify leaf‐thickening development at the cellular level.

**Figure 7:**
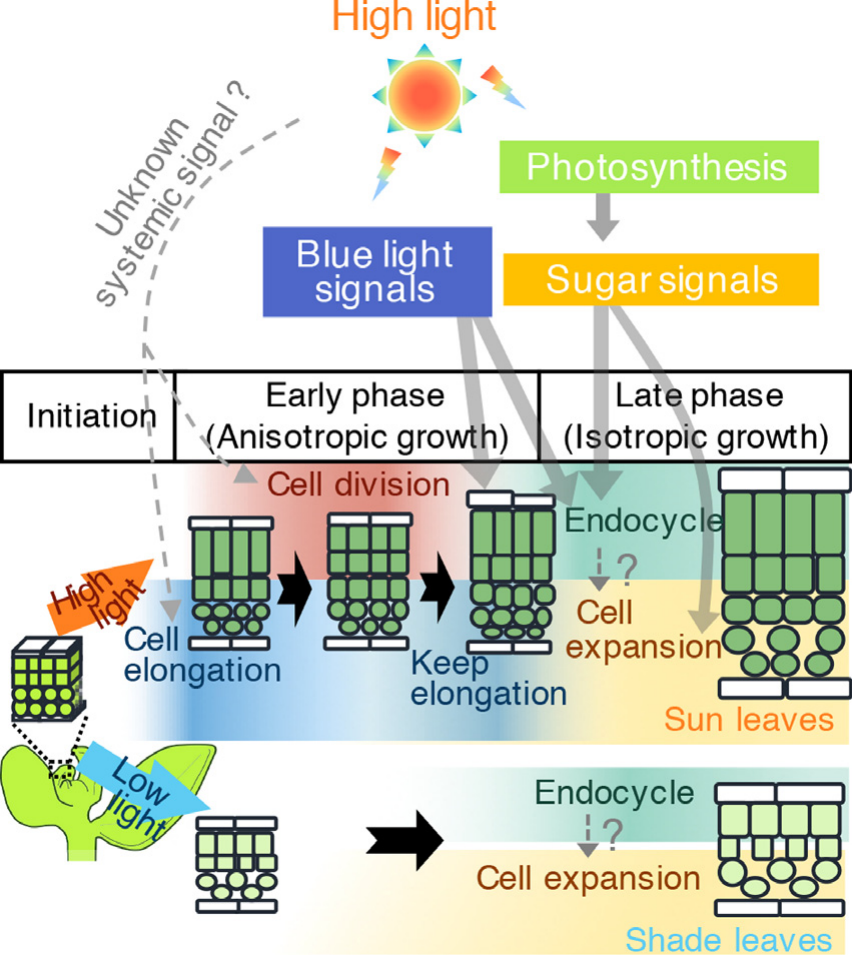
Developmental framework of leaf thickening in sun and shade leaves. Diagrams represent cross‐sectional images of leaves at each developmental stage. A detailed explanation is provided in the discussion section in the text. Upper and lower halves show sun‐ and shade‐leaf formation, respectively. Leaf development progresses from left to right. Bold black arrows indicate developmental steps, and thin grey arrows indicate the effects of sugar and blue‐light signals from cryptochromes and phototropins on leaf development. Dotted lines indicate the hypothetical pathways.

There are some concerns about observing young leaf primordia using conventional sectioning methods. For example, the considerably small size of the sample can easily result in improper positioning of the sample against the microtome, resulting in oblique sections and incorrect cell measurement. Additionally, cell size and cell layer numbers differ along the leaf longitudinal axis and at the margins of leaf primordia; thus, both longitudinal and transverse sections are needed to confirm morphological changes at the cellular level (Figure S2c; Donnelly *et al*., 1999). To facilitate the description of leaf‐thickness growth, we modified an observation method and succeeded in obtaining a wide range of serial longitudinal sections in semi‐whole‐mount samples (Figure S2a–c). This method enabled us to clearly analyse cell shapes and numbers, both in the dorsoventral direction and in the paradermal plane, and even to characterize the morphology of tiny leaf primordia at 4 DAS, which were just emerging from the shoot apical meristem, and which have not previously been analysed in detail (Figures 2a and S2a–c). Based on these observations, we found that the initial structure of leaf internal tissue in leaf primordia before 5 DAS was indistinguishable between seedlings grown under HL and LL conditions (Figures 2a and 7). Kalve *et al*. (2014) also analysed leaf thickening in HL‐ and LL‐grown seedlings and proposed that sun or shade leaves are determined immediately after germination; however, they observed leaf transverse structures from 5 DAS onwards and lacked observations of earlier stages, presumably because of the technical limitations of conventional plastic sectioning, as discussed above. In this study, our optimized technique enabled the detailed observation of the internal structure of early leaf primordia and led us to conclude that sun and shade leaves are morphologically indistinguishable until 4 DAS. Similar to our observations, identical internal structures between sun‐ and shade‐leaf primordia were observed in *C. album* (Yano and Terashima, 2004), suggesting that sun and shade leaves differentiate from the same type of leaf primordia over the course of development. From further observations, we characterized the stepwise developmental processes of sun‐leaf formation in Arabidopsis (Figures 2a and 7). At the early phase, cell elongation and subsequent periclinal cell division along the dorsoventral direction occurred in the palisade tissue, and these processes resulted in anisotropic leaf‐thickening growth under HL conditions. Subsequently, isotropic cell expansion occurred, and intercellular space increased during the late stage of leaf thickening in both sun‐ and shade‐leaf formation (Figure 7).

### Effects of blue‐light receptors on sun‐leaf formation

Anisotropic cell elongation in the palisade tissue is the first unique step of sun‐leaf formation (Figure 2a). Blue light reportedly promotes the anisotropic cell elongation of palisade tissue in the dorsoventral direction of the leaf, but mutants lacking either class of blue‐light receptors do not display defects in sun‐leaf formation (Weston *et al*., 2000; López‐Juez *et al*., 2007). Nevertheless, under blue light of moderate intensity (100 μmol photons m^−2^ s^−1^ in photosynthetic photon flux density), the *phot1‐5 phot2‐1* mutant was reported to have defects in the formation of cylindrical palisade cells (Kozuka *et al*., 2011). Thus, their results were inconsistent. To re‐examine this matter, we employed two allelic combinations of the phototropin double mutants *phot1‐5 phot2‐1* and *phot1‐5 phot2‐2*, both of which were demonstrated as null‐allele mutants in previous studies (Figures 4 and S4; Kozuka *et al*., 2011; Suetsugu *et al*., 2013). Here, we found that loss of phot1 and phot2 activity did not lead to the complete loss of anisotropic cell elongation in the palisade tissue under white HL conditions (Figures 4a and S4). Thus, our results suggest that phototropins are not exclusively required for anisotropic cell elongation, at least under our white HL conditions, as described by López‐Juez *et al*. (2007).

We also examined whether the cell shape of palisade tissue was determined by blue‐light irradiation (Figure S5). Under strong blue light, wild‐type seedlings formed cylindrically elongated cells in the palisade tissue. Moreover, anisotropic cell elongation was reduced in blue‐light receptor mutants (Figure S5). The *cry1 cry2* mutant showed smaller cells through a reduction in cell height, whereas the *phot1 phot2* mutant changed the orientation of cell elongation from the dorsoventral to the paradermal direction, but did not show a reduction in cell size in response to strong blue light (Figure S5a,d,f). Therefore, both phototropins and cryptochromes could possibly mediate anisotropic cell elongation in response to strong blue light during sun‐leaf formation.

### Defects in anisotropic cell elongation in the *cry1 cry2 phot1 phot2* quadruple mutant

We also found that, even under strong white‐ and blue‐light conditions, only the *cry1 cry2 phot1 phot2* quadruple mutant showed reduced leaf thickness through decreased anisotropic cell elongation in palisade cells (Figures 4a and S5). During leaf development, anisotropic cell elongation occurred in the *cry1 cry2 phot1 phot2* quadruple mutant as well as in the wild type at 5 DAS, but anisotropic cell elongation after periclinal cell divisions from 6 DAS was absent in the quadruple mutant under HL conditions (Figure 5a,c,e). This indicates that the two types of anisotropic cell elongation are regulated by blue‐light receptor‐dependent and ‐independent pathways before and after periclinal cell division, respectively (Figure 5).

It is generally understood that the signalling pathways of cryptochromes and phototropins do not interact (Ohgishi *et al*., 2004). Some blue‐light responses, such as hypocotyl growth, stomatal opening and chloroplast movement, are thought to be independently mediated by cryptochromes and phototropins (Ohgishi *et al*., 2004; Boccalandro *et al*., 2012). Here, we revealed that phototropins and cryptochromes mediate the determination of palisade cell shape in response to HL conditions (Figures 4 and S5). In terms of palisade cell shape, the *cry1 cry2* *phot1 phot2* quadruple mutant showed additive phenotypes of the two respective double mutants (*cry1 cry2* and *phot1 phot2*) under blue‐light but not under red‐light conditions (Figures 4 and S5), suggesting that the canonical functions of cryptochromes and phototropins as blue‐light receptors are required for anisotropic cell elongation. The function of either cryptochromes or phototropins was sufficient to induce the palisade phenotype under white HL conditions, however, but not under the strong blue‐light conditions (Figures 4 and S5). Therefore, in addition to blue‐light sensing, another mechanism is involved in the determination of palisade cell phenotype in response to white HL conditions. Generally, white HL conditions generated using fluorescent lamps includes light of wavelengths 400–750 nm (López‐Juez *et al*., 2007; Pashkovskiy *et al*., 2016). The canonical functions of blue‐light receptors are modified under some light conditions. For example, hypocotyl elongation is repressed under blue light and this response is mediated by cryptochromes, whereas irradiation with both blue and green light inhibits blue light‐dependent repression of hypocotyl elongation (Wang *et al*., 2013). The active form of cryptochromes can be converted into the inactive form by further perception of green light (Bouly *et al*., 2007). In addition, irradiation with white fluorescent light increases the transcript levels of several genes, including photoreceptors, auxin‐response factors, and miRNAs, compared with the transcript levels under blue light of identical intensity (Pashkovskiy *et al*., 2016).

### Effect of sugar signalling on sun‐leaf formation

Sugars are highly produced under HL conditions by enhanced photosynthesis and translocated from mature (source) leaves to the shoot apex (sink), and activate cell proliferation in leaf primordia (Wang and Nobel, 1996; Khamlichi *et al*., 2000; Jänkänpää *et al*., 2012). Also, thick leaves were formed in some transgenic Solanaceae species or Arabidopsis mutants in which altered sugar metabolism or transport result in the high accumulation of soluble sugars in leaves (Hoffmann‐Benning *et al*., 1997; Keller *et al*., 1998; Zobayed *et al*., 1999; Wuyts *et al*., 2012). These studies suggest a correlation between sugars and leaf thickening, and sugars potentially meet the criteria for a systemic signal that controls the formation of sun and shade leaves; however, the effect of sugars on leaf thickening in sun‐leaf formation was unknown in Arabidopsis. For these reasons, we fed LL‐ and HL‐grown seedlings with sucrose.

We found that this exogenous treatment slightly affected leaf‐thickness growth by modulating both cell height and paradermal cell area, which resulted from the enhancement of isotropic cell expansion (Figures 6d,e and S4), whereas periclinal cell division was seen exclusively under HL conditions (Figure 6b). Thus, sucrose can promote the late phase of leaf thickening, but may not be involved in the early phase of sun‐leaf formation in Arabidopsis. Furthermore, both the endocycle and mitotic cycle were upregulated in a sucrose concentration‐ and light intensity‐dependent manner (Figure 6c,f). Statistical tests also detected an interaction between the effects of sucrose treatment and light conditions on leaf area, but not on leaf thickness (Table S1). Therefore, sucrose regulates cell division and expansion to control leaf paradermal growth. These data also indicate that another important factor is likely to be involved in the induction of periclinal cell division in response to HL conditions.

### Leaf thickening is regulated in endocycle‐dependent and ‐independent manners

Cell enlargement often occurs concomitantly with increases in ploidy levels during organogenesis in some plant species (Traas *et al*., 1998; Kondorosi *et al*., 2000; Sugimoto‐Shirasu and Roberts, 2003). Regarding leaf‐thickness growth, corresponding increases in cell size and ploidy levels were reported in Solanaceae species (Gauhl, 1976; Coneva *et al*., 2017). In a recent study, the light‐shift experiment in Arabidopsis showed that exposure to HL conditions promoted the endocycle in Arabidopsis (Mohammed *et al*., 2018). Several exceptions to the correlation of cell size with endocycle progression have been reported in Arabidopsis leaves, however (Gendreau *et al*., 1998; Tsukaya, 2008; Katagiri *et al*., 2016). To elucidate the effects of endocycle progression on cell morphogenesis in sun‐leaf formation, we traced the process of development in sun‐ and shade‐leaf primordia (Figure 2a–f). From 5 to 7 DAS, cell sizes in all layers of mesophyll tissue were apparently larger under HL than under LL conditions, whereas ploidy levels only differed slightly between the light conditions (Figures 2a,b, 5c, and S1b). In addition, the *bin4* mutant with an endocycle defect still formed anisotropically elongated cells under HL conditions (Figure 3a,b,a′,b′). Conversely, even shade leaves showed a gradual increase in ploidy levels, especially in the late phase of leaf development, although such endocycle progression did not accompany anisotropic cell elongation (Figures 2a,b and 6d,f). These results indicate that the anisotropic growth of palisade cells does not require endocycle progression; the morphological differentiation of sun and shade leaves is unlikely to be induced by endocycle activation.

Leaf thickness is finalized by isotropic cell expansion and an increase in intercellular space during the late phase of leaf development (Figure 2a). This phase was observed irrespective of sun‐ or shade‐leaf formation and was associated with endocycle progression (Figure 2b). Additionally, the final cell size and ploidy levels were reduced in mutants defective in endocycle progression and blue‐light receptors (Figures 3 and 4). Consequently, isotropic cell expansion may require an increase in ploidy levels.

## Conclusion

We propose a model of leaf thickening in sun and shade leaves, as illustrated in Figure 7. Sun and shade leaves are initially indistinguishable at the very young stage of leaf primordia development (i.r. the ‘initial phase’, before 5 DAS; Figure 7). Upon sensing the light intensity by existing leaves or cotyledons, leaf primordia differentiate into shade or sun leaves. During the first step of sun‐leaf formation under HL conditions, anisotropic cell elongation and periclinal cell division occur in the palisade tissue to increase leaf thickness. Subsequently, divided cells again undergo anisotropic cell elongation, and this second cell elongation after periclinal cell division is controlled by blue light via phototropins and cryptochromes. The two types of anisotropic cell elongation, together with periclinal cell divisions, are categorized as anisotropic growth, which occurs at the ‘early phase’, i.e. 5–7 DAS (Figure 7). Finally, all leaf mesophyll cells isotropically expand to increase both leaf thickness and leaf area during the ‘late phase’ after periclinal cell divisions from 8 DAS onwards (Figure 7). This isotropic cell expansion is activated by sugar‐dependent signal(s). In contrast to sun‐leaf formation, shade‐leaf formation skips anisotropic growth in the early phase and involves only the late phase of leaf thickening. Given that the late phase was shared by both sun‐ and shade‐leaf formation, isotropic growth in the late phase is likely to be the default programme for leaf‐thickness growth in Arabidopsis. These multiple steps of leaf thickening in response to different cues may provide a flexible system for adjusting leaf structure at different stages of leaf development, and may enable the optimization of photosynthesis in response to surrounding light intensity, as the light source is an essential but unstable factor in the field environment.

## Experimental procedures

### Plant materials

Arabidopsis Columbia‐0 (Col‐0) or Col‐*gl1* (a *glabra1‐S92F* mutation in a Columbia background) was used as the wild type in this study. The following mutants and transgenic marker lines in the Col‐0 background have been described previously: *bin4‐1* (Yin *et al*., 2002), *cry1‐304 cry2‐1* (Mockler *et al*., 1999), *phot1‐5 phot2‐1 gl1* and *phot1‐5 phot2‐2 gl1* (Kinoshita *et al*., 2001; Kozuka *et al*., 2011) and *pATML1::H2B‐mGFP* (Roeder *et al*., 2010). The *cry1‐304 cry2‐1 phot1‐5 phot2‐1* *gl1* mutant was generated by crossing the *cry1‐304 cry2‐1* mutant with the *phot1‐5 phot2‐1 gl1* mutant (Kinoshita *et al*., 2001), which showed identical phenotype to *phot1-5 phot2-2 gl1* (Figure S4). For clarity, we term this multiple mutant the *cry1 cry2 phot1 phot2* quadruple mutant, because the *gl1* mutation did not influence the morphology of the leaves or the cells (Figure S1c–g). For all analyses we used *cry1‐304 cry2‐1 phot1‐5 phot2‐2 gl1* in this report, except in Figure S4.

### Growth conditions

In the laboratory conditions, the standard light intensity for Arabidopsis culture is usually set at 50–150 μmol photons m^−2^ s^−1^ in photosynthetic photon flux density. These light conditions induce shade leaves with poorly developed palisade tissue. To induce a clear difference in leaf thickness between sun and shade leaves, here we grew plants under HL (280 μmol m^−2^ s^−1^) or LL (70 μmol m^−2^ s^−1^) conditions in a growth chamber (LH‐350S; NK‐systems, Japan, http://www.nksystems.jp) under a light/dark cycle of 16/8 h at 22°C (Figure S8). Light intensity was measured using a light meter (LI‐250A; Li‐Cor, https://www.licor.com; LA‐105; NK‐systems).

Seeds were sterilized in plant preservative mixture solution (PPM; Nacalai Tesque, https://www.nacalai.co.jp) and sown on MS medium plates (0.5× Murashige and Skoog salts and 0.5% w/v gellan gum, pH 5.8) or rockwool (M35T40; Nittobo, https://www.nittobo.co.jpJapan), as described previously (Tsukaya *et al*., 1991; Minamisawa *et al*., 2011).

For sucrose treatment, surface‐sterilized seeds were sown on MS plates with sucrose concentrations ranging from 0.1 to 2% (w/v).

### Observation of leaf and cell morphology

We used the modified pseudo‐Schiff propidium iodide (mPS‐PI) method (Ichihashi *et al*., 2011) to visualize leaf longitudinal sections of young leaf primordia (Figure S2a–c). Young seedlings were harvested at 4–8 DAS and fixed in a solution of 50% methanol and 10% acetic acid. As shown in Figure S2(a), cotyledons were removed from fixed samples, then both sides of the leaf primordia were trimmed before staining with mPS‐PI. Samples were cleared using the transparent plant organ method for imaging (TOMEI) or chloral hydrate solution (Tsuge *et al*., 1996; Hasegawa *et al*., 2016), and were placed on glass slides and covered with coverslips, with the cut side of the leaf primordia facing the microscope lens. These samples were observed using confocal microscopy (model LSM 710; Carl Zeiss, https://www.zeiss.com). This allowed us to observe clear images of optical leaf longitudinal sections up to 200 μm in depth. We obtained *z*‐sections at 1–3 μm intervals at the middle part of the primordia and used these for morphological measurements (Figures 2c and 5a–e). To observe mature leaves, the first pairs of fully expanded foliage leaves at 21 DAS were fixed in FAA solution (5% acetic acid, 45% ethanol and 5% formaldehyde) overnight or longer (Tsukaya *et al*., 1993). To observe transverse sections, fixed leaves were dehydrated in a graded ethanol series and embedded in Technovit 7100 resin (Kulzer, https://www.kulzer.com). Sections of 3‐ to 6‐μm in thickness were placed on coverslips, stained with 0.1% (w/v) toluidine blue in 0.1 M phosphate buffer (pH 7.0), dried, and mounted on glass slides with Entellan new mounting medium (EMD Millipore, now Merck, http://www.merckmillipore.com). To observe optical paradermal sections, fixed samples were treated with chloral hydrate solution overnight, and a stereoscopic microscope (MZ16; Leica, Germany, http://www.leicabiosystems.com) and a differential interference contrast microscope (DM4500; Leica) were used. For leaf shape measurement, fixed leaves with slits in the leaf margin were extended and flattened on glass slides and then scanned with a scanner (GT‐X900; Epson, Japan, https://epson.com).

### Measurement of leaf and cell morphology

All morphological measurements of leaves and cells were performed using imagej fiji (https://fiji.sc/). The adaxial subepidermal layer cells (the first layer of palisade mesophyll tissue) in the middle of leaf blades were measured to quantify the representative cell shape of palisade tissue in the resin sections or the series of optical sections. The leaf thickness of individual samples was calculated as the mean of four distinct points in cross section (excluding the leaf vein and margin). Cell height, width and area were measured as the cell length of a vertical or a horizontal line to the surface or outline of the cells. These values were calculated as the mean of more than 60 cells from between three and five plants. The total number of cells in the palisade mesophyll tissue per leaf was calculated by multiplying the leaf area, cell density in the paradermal plane and average number of cell layers in the palisade mesophyll tissue; the means of these parameters are listed in Table S2.

### Flow cytometry

The ploidy levels of leaf blades were quantified by flow cytometry (BD FACS AriaII or Accuri C6; Becton Dickinson, https://www.bd.comUSA) as described previously (Kozuka *et al*., 2005). Approximately 10^3^–10^4^ cells were measured per sample. All results are presented as the mean of three independent measurements or representative data from at least three independent biological replicates.

### Statistical analysis

prism 6.0 (GraphPad, https://www.graphpad.com) was used for statistical analyses. A Student's *t*‐test was used to compare the mean cell area, height and the number of cell layers between the two groups (sun and shade leaves induced in rockwool). To determine the effects of light intensity and sucrose concentration on leaf growth, data were statistically analysed using two‐way anova with Tukey's *post hoc* test for all pairwise comparisons. Detailed results are shown in Table S1.

## Data statement

All data related to this paper can be found within the manuscript and supplementary files.

## Conflict of interest

We have no conflicts of interest to declare.

## Authors contributions

RH and HT designed the research plan; RH performed all experiments and analysed the data; YY generated the *cry1 cry2 phot1 phot2* quadruple mutant; and RH, YY and HT wrote the article.

## Supporting information


**Figure S1.** Cell morphology and ploidy levels of the epidermal and mesophyll cells of sun and shade leaves.
**Figure S2.** Method developed for observing longitudinal sections of young leaf primordia.
**Figure S3.** Effect of sucrose on leaf morphology in the *bin4* mutant.
**Figure S4.** Leaf morphology of the *phot1‐5 phot2‐1* double mutant under HL conditions.
**Figure S5.** Leaf morphology of blue‐light receptor mutants under monochromatic blue‐ and red‐light conditions.
**Figure S6.** Effect of sucrose on cell morphology in the wild type.
**Figure S7.** Effects of osmotic pressure and sucrose concentration on cell size in the wild type.
**Figure S8.** Spectral photon irradiance of the light.

**Table S1.** Statistical data on the anatomical parameters of wild‐type plants exogenously supplied with sucrose.
**Table S2.** Mean anatomical parameters of wild‐type plants exogenously supplied with sucrose.
