# Supplementary material for: Multiple steps of leaf thickening during sun‐leaf formation in Arabidopsis
Source: Plant J. 2019 Sep 9;100(4):738–53. doi: 10.1111/tpj.14467 (PMC6900135; doi:10.1111/tpj.14467)
Supplement: Supplementary file 1 — Figure S1. Cell morphology and ploidy levels of the epidermal and mesophyll cells of sun and shade leaves. Figure S2. Method developed for observing longitudinal sections of young leaf primordia. Figure S3. Effect of sucrose on leaf morphology in the bin4 mutant. Figure S4. Leaf morphology of the phot1‐5 phot2‐1 double mutant under HL conditions. Figure S5. Leaf morphology of blue‐light receptor mutants under monochromatic blue‐ and red‐light conditions. Figure S6. Effect of sucrose on cell morphology in the wild type. Figure S7. Effects of osmotic pressure and sucrose concentration on cell size in the wild type. Figure S8. Spectral photon irradiance of the light. [file TPJ-100-738-s001.docx]

**Supporting Figures**

**
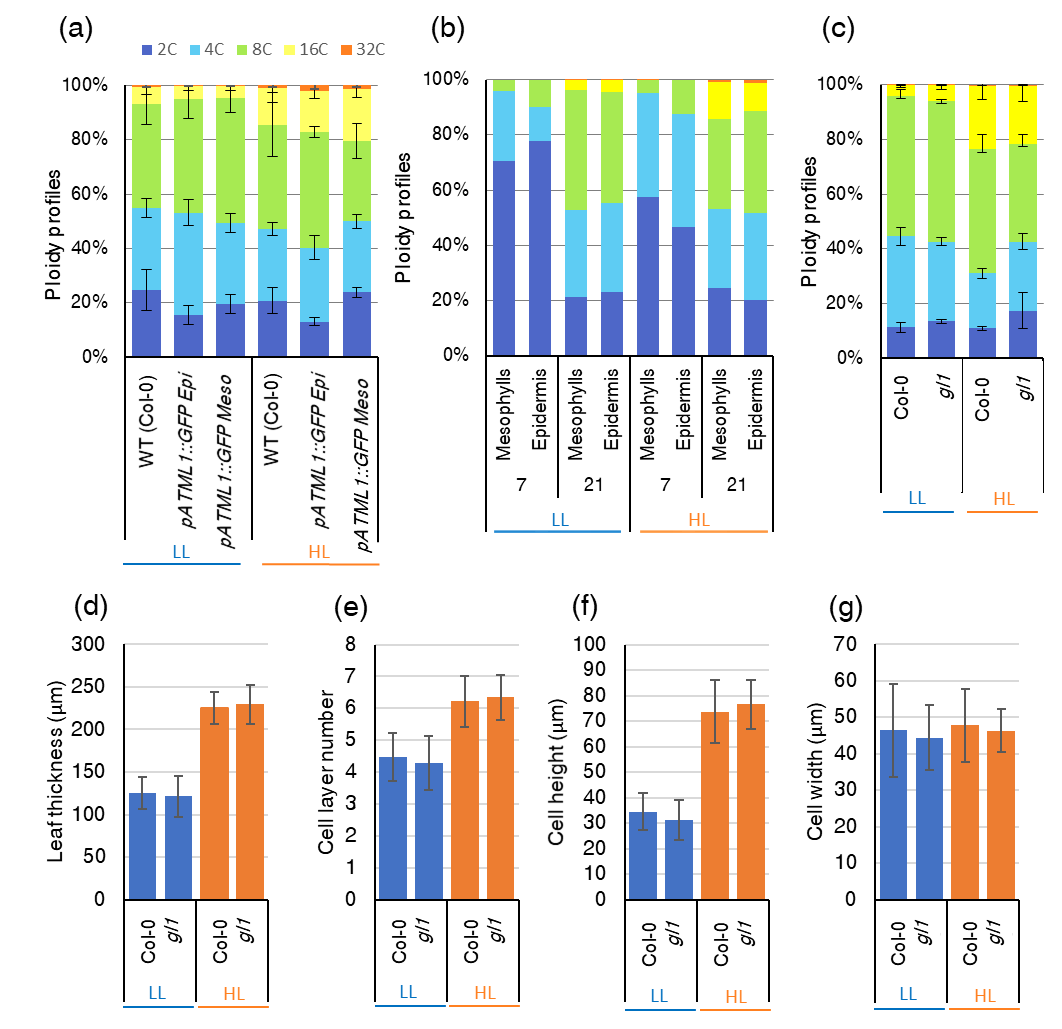
**

**Figure S1.** Ploidy levels of the epidermal and mesophyll cells in sun and shade leaves.

(a) Ploidy levels of seedlings of wild-type (Col-0) and *pATML1::H2B-mGFP* transgenic Arabidopsis at 21 days after sowing (DAS) under low light (LL) and high light (HL) conditions. Values represent the mean ± SD (n = 6 leaves from three plants). (b) Ploidy levels of developing (7 DAS) and mature (21 DAS) sun and shade leaves in *pATML1::H2B-mGFP* transgenic plants. Owing to the small size of 7-DAS leaves, ploidy measurement required more than 10 leaves per sample (n = 10 leaves from five plants). The ploidy profiles of epidermal (Epi) or mesophyll (Meso) tissue cells in the same leaf samples were separately counted from positive or negative fluorescence of *pATML1:: H2B-mGFP*, respectively*.* (c) Ploidy levels of two control lines used in this study (*gl1* in Figure 4, 5, S4, S5; Col-0 in the other Figures). Note that, both lines have same ploidy levels under LL and HL conditions, respectively. (d-g) Comparison of sun and shade leaf formation in the control lines: Col-0 and Col-*gl1*. (d) Leaf thickness, (e) cell layer number, (f) palisade cell height, and (g) palisade cell width. Values represent mean ± SD (n = four leaves from different individuals). We performed Student’s t-test to compare each combination (Col-0 vs *gl1* in each light condition), but there were no significant differences (P < 0.0001).

**
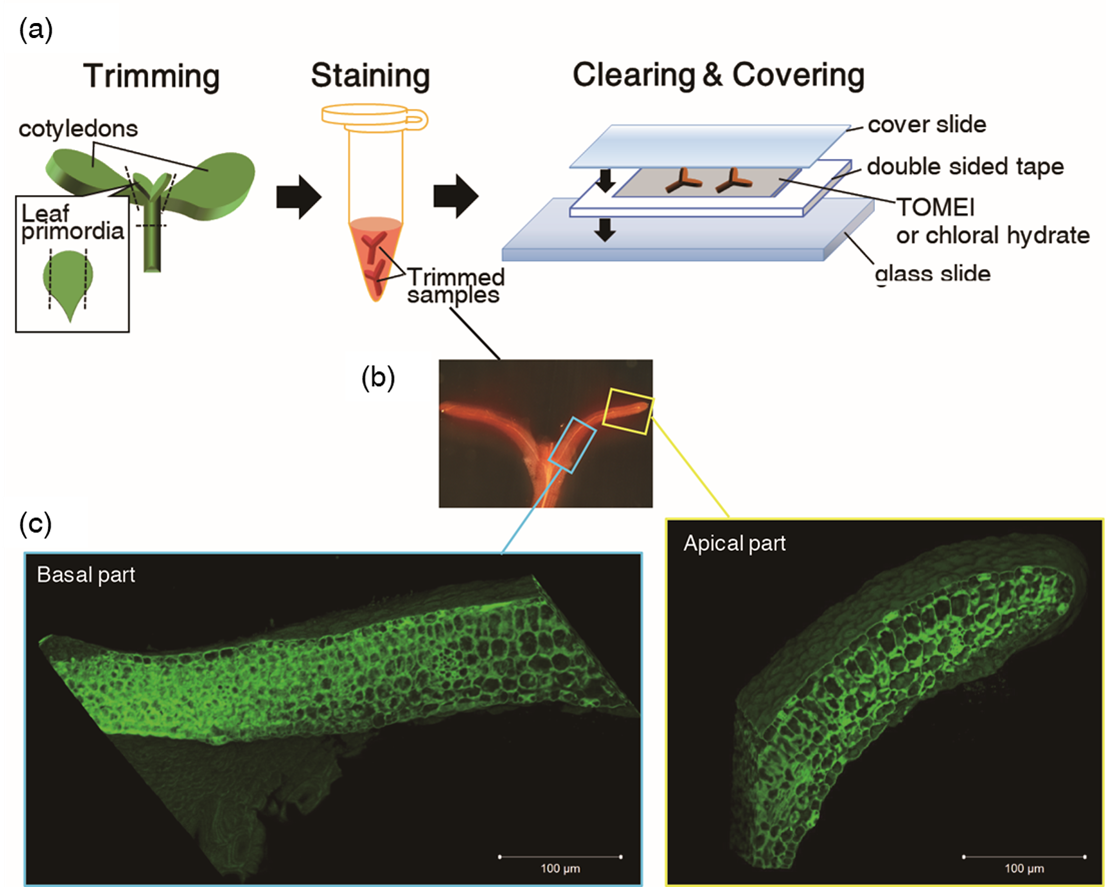
**

**Figure S2.** Developed method for observing longitudinal sections of young leaf primordia.

(a) Graphical overview of sample preparation. (b) A 7-DAS seedling after sample preparation. (c) Confocal images of leaf primordia obtained by this method (left: basal part; right: apical part). Note that the tissue structure (cell size, shape, and cell layer numbers) differed depending on the position of the leaf primordia. Cell walls were stained with modified pseudo-Schiff propidium iodide (mPS-PI) and observed under a confocal microscope (excitation: 488 nm, emission: 570–700 nm). Detailed methods are described in the Experimental procedures section in the text.

**
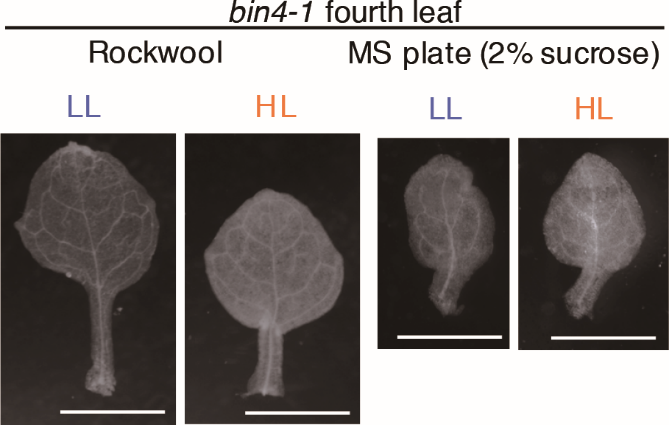
**

**Figure S3.** Effect of sucrose on leaf morphology in the *bin4* mutant.

Largest (fourth) foliage leaves of *bin4* mutants grown on rockwool (left) and MS plates with 2% sucrose (right) under LL and HL conditions. *Scale bars* = 1 mm.

**
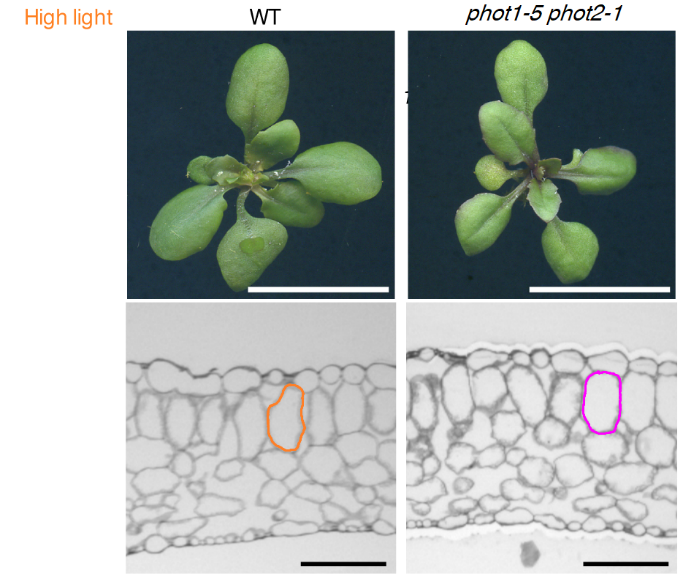
**

**Figure S4.** Leaf morphology of the *phot1-5 phot2-1* double mutant under HL condition.

Wild-type (Col-*gl1*) and double mutant plants lacking phototropins (*phot1-5 phot2-1*), which are thought to be null alleles, were cultivated under HL for 18 days. Rosette morphology (upper panels) and leaf cross sections (lower panels) of the first pair of foliage leaves of each genotype. Typical mesophyll tissue cells are outlined at each stage. At least three individuals per genotype were observed. *Scale bars:* white bars = 1 cm, black bars = 100 μm.

**
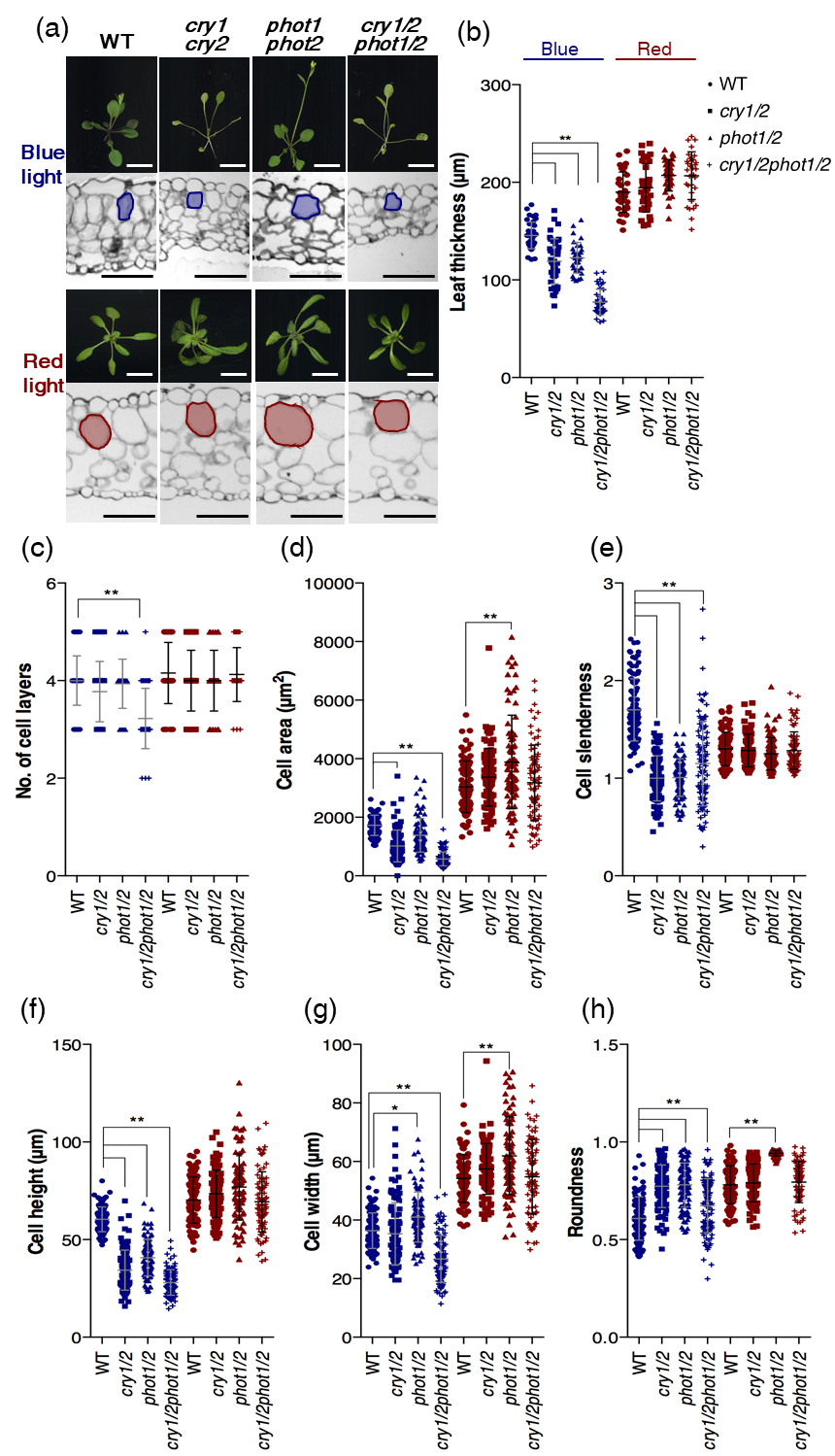
**

**Figure S5.** Morphology of blue-light receptor mutants under monochromatic blue- and red-light conditions.

Wild-type (WT, *gl1*) and mutant plants with defects in blue-light sensing, *phot1 phot2* double mutant (*phot1/2*), *cry1 cry2* double mutant (*cry1/2*), and *cry1 cry2 phot1 phot2* quadruple mutant (*cry1/2 phot1/2*), were cultivated under blue and red LED in 200 μmol photons m^-2^ s^-1^for 21 days. (a) Rosette morphology (upper panels) and leaf cross-sections (lower panels) of the first pair of foliage leaves in each genotype. Typical mesophyll tissue cells are outlined. (b) Leaf thickness (c) cell layer number, (d) palisade cell area, (e) cell slenderness (cell height/ cell width), (f) cell height, (g) cell width, and (h) roundness. Values represent the mean ± SD (n > four to five plants). *Scale bars:* white bars = 1 cm, black bars = 100 μm. Asterisks indicate statistically significant differences by Sidak’s multiple comparison test (*P* < 0.0001: **, 0.001 < *P* < 0.01: *).

**
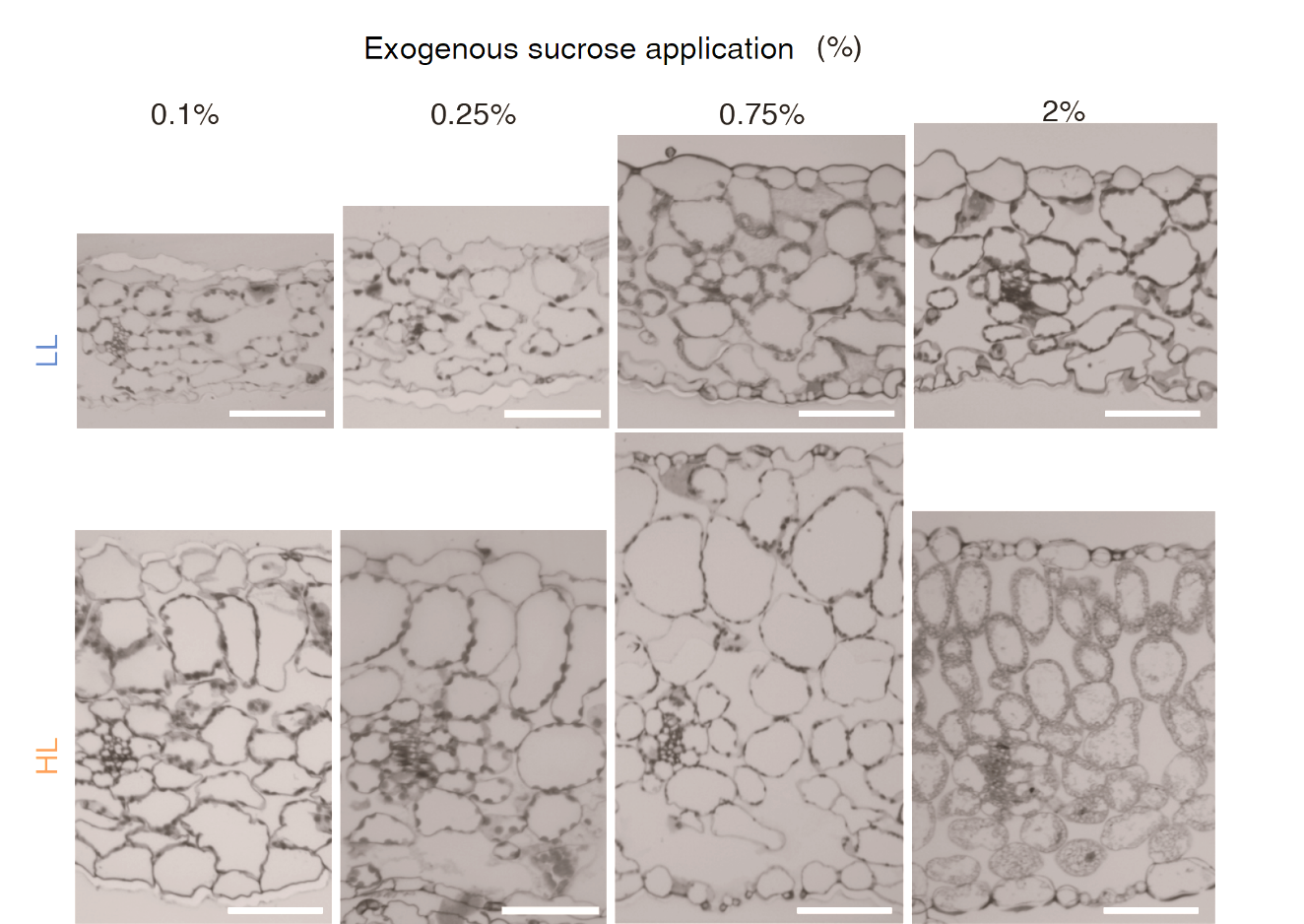
**

**Figure S6.** Effect of sucrose on cell morphology in the wild type.

Leaf sections were made from the first pair of foliage leaves of wild-type seedlings grown on MS plates with exogenous sucrose application under LL and HL conditions. *Scale bars* = 100 μm.


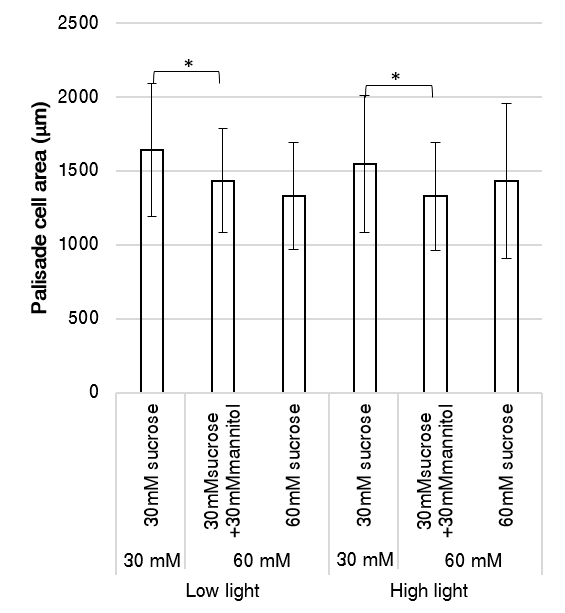


**Figure S7.** Effects of osmotic pressure and sucrose concentration on cell size in the wild type.

In this experiment, mannitol, an unmetabolized sugar, was used as a control for osmotic effects. Wild type (Col-0) plants were cultivated on three plate conditions with exogenous sugars: 30 mM sucrose, 60 mM sucrose or 30 mM sucrose with 30 mM mannitol (totally 60 mM). Cell size in the first layer of palisade cells was measured from paradermal images. Values represent mean ± SD (n = three leaves). Asterisks indicate statistical differences (*P* < 0.001).

**Figure S8**. Spectral photon irradiance of the light.

Light intensity between 380-700 nm at all light conditions used in study. HL: high light condition 280 μmol m^-2^ s^-1^, BL: blue light (200 μmol m^-2^ s^-1^ ), RL: red light (200 μmol m^-2^ s^-1^), and solar spectra measured at sunny (1674 μmol m^-2^ s^-1^ ), mostly sunny (1177 μmol m^-2^ s^-1^ ) and cloudy day (357 μmol m^-2^ s^-1^ ). Summing over the range of 400-700 nm yields, PPFD (photosynthesis photon flux density) of these solar spectra are described in parentheses.
