# Supplementary material for: Multiple steps of leaf thickening during sun‐leaf formation in Arabidopsis
Source: Plant J. 2019 Sep 9;100(4):738–53. doi: 10.1111/tpj.14467 (PMC6900135; doi:10.1111/tpj.14467)
Supplement: Supplementary file 2 — Table S1. Statistical data on the anatomical parameters of wild‐type plants exogenously supplied with sucrose. Table S2. Mean anatomical parameters of wild‐type plants exogenously supplied with sucrose. [file TPJ-100-738-s002.docx]

**SUPPLEMENTAL INFORMATION - Tables**

| **Leaf area (μm^2^)** |  |  |  |  |  |
| --- | --- | --- | --- | --- | --- |
| ANOVA table | SS | DF | MS | F (DFn, DFd) | P value |
| Interaction | 3.30E+13 | 3 | 1.10E+13 | F (3, 16) = 1.893 | **P = 0.1715** |
| Row Factor | 1.94E+15 | 3 | 6.46E+14 | F (3, 16) = 111.1 | **P < 0.0001** |
| Column Factor | 1.92E+14 | 1 | 1.92E+14 | F (1, 16) = 33.03 | **P < 0.0001** |
| Residual | 9.30E+13 | 16 | 5.81E+12 |  |  |
| **No. of cells per leaf** |  |  |  |  |  |
| ANOVA table | SS | DF | MS | F (DFn, DFd) | P value |
| Interaction | 1.12E+08 | 3 |  | F (3, 16) = 1.582 | P = 0.2328 |
| Row Factor | 1.70E+09 | 3 | 5.67E+08 | F (3, 16) = 24.12 | P < 0.0001 |
| Column Factor | 3.34E+05 | 1 | 3.34E+05 | F (1, 16) = 0.01419 | P = 0.9067 |
| Residual | 3.76E+08 | 16 | 2.35E+07 |  |  |
| **Palisade cell area (μm^2^)** |  |  |  |  |  |
| ANOVA table | SS | DF | MS | F (DFn, DFd) | P value |
| Interaction | 2308000 | 3 | 769194 | F (3, 952) = 5.659 | P = 0.0008 |
| light | 73560000 | 3 | 24520000 | F (3, 952) = 180.4 | P < 0.0001 |
| sucrose | 11130000 | 1 | 11130000 | F (1, 952) = 81.85 | P < 0.0001 |
| Residual | 129400000 | 952 | 135923 |  |  |
| **Palisade cell height (μm)** |  |  |  |  |  |
| ANOVA table | SS | DF | MS | F (DFn, DFd) | P value |
| Interaction | 2904 | 3 | 968.1 | F (3, 472) = 5.383 | P = 0.0012 |
| light | 7067 | 3 | 2356 | F (3, 472) = 13.10 | P < 0.0001 |
| sucrose | 351821 | 1 | 351821 | F (1, 472) = 1956 | P < 0.0001 |
| Residual | 84886 | 472 | 179.8 |  |  |
| **Leaf thickness (μm)** |  |  |  |  |  |
| ANOVA table | SS | DF | MS | F (DFn, DFd) | P value |
| Interaction | 22557 | 3 | 7519 | F (3, 88) = 6.011 | P = 0.0009 |
| light | 76420 | 3 | 25473 | F (3, 88) = 20.37 | P < 0.0001 |
| sucrose | 794498 | 1 | 794498 | F (1, 88) = 635.2 | P < 0.0001 |
| Residual | 110072 | 88 | 1251 |  |  |
| **Cell density (cells/μm^2^)** |  |  |  |  |  |
| ANOVA table | SS | DF | MS | F (DFn, DFd) | P value |
| Interaction | 2.935E-08 | 3 | 9.783E-09 | F (3, 16) = 1.818 | P = 0.1845 |
| Row Factor | 2.542E-07 | 3 | 8.473E-08 | F (3, 16) = 15.74 | P < 0.0001 |
| Column Factor | 3.638E-08 | 1 | 3.638E-08 | F (1, 16) = 6.758 | P = 0.0194 |
| Residual | 8.612E-08 | 16 | 5.383E-09 |  |  |
| **No. of palisade cell layer** |  |  |  |  |  |
| ANOVA table | SS | DF | MS | F (DFn, DFd) | P value |
| Interaction | 1.021 | 3 | 0.3403 | F (3, 16) = 1.543 | P = 0.2419 |
| Row Factor | 3.095 | 3 | 1.032 | F (3, 16) = 4.679 | P = 0.0157 |
| Column Factor | 9.796 | 1 | 9.796 | F (1, 16) = 44.43 | P < 0.0001 |
| Residual | 3.528 | 16 | 0.2205 |  |  |

**Supplemental Table 1.** Statistical data on the anatomical parameters of wild-type plants exogenously supplied with sucrose.

The effects of light intensity and sucrose concentration were analysed using two-way analysis of variance (ANOVA) for all pairwise comparisons.

|  | Mean of palisade cell density in the paradermal direction (No. cells/μm^2^) | Leaf area (mm^2^) | Mean of number of cells in palisade tissue layers (calculated from 4 points per leaf) |
| --- | --- | --- | --- |
| HL0.1%-1 | 551.7 | 6 | 2 |
| HL0.1%-2 | 423.7 | 5 | 2 |
| HL0.1%-3 | 711.6 | 5 | 2 |
| HL0.25%-1 | 649.2 | 14 | 2 |
| HL0.25%-2 | 672.7 | 15 | 2 |
| HL0.25%-3 | 622.3 | 14 | 2 |
| HL0.75%-1 | 501.2 | 24 | 2 |
| HL0.75%-2 | 605.4 | 27 | 2 |
| HL0.75%-3 | 433.9 | 29 | 3 |
| HL2%-1 | 541.5 | 19 | 3 |
| HL2%-2 | 682.8 | 22 | 3 |
| HL2%-3 | 551.6 | 26 | 2 |
| LL0.1-1 | 535.7 | 11 | 2 |
| LL0.1-2 | 511.7 | 11 | 1 |
| LL0.1-3 | 559.7 | 9 | 2 |
| LL0.25-1 | 918.3 | 18 | 2 |
| LL0.25-2 | 571.8 | 16 | 2 |
| LL0.25-3 | 713.1 | 17 | 2 |
| LL0.75-1 | 568.4 | 35 | 1 |
| LL0.75-2 | 521.4 | 41 | 2 |
| LL0.75-3 | 464.2 | 32 | 2 |
| LL2%-1 | 706.4 | 26 | 2 |
| LL2%-2 | 713.1 | 30 | 1 |
| LL2%-3 | 713.1 | 29 | 2 |

**Supplemental Table 2.** Mean anatomical parameters of wild-type plants exogenously supplied with sucrose.

Mean parameters calculated from measurements of individual plants (three plants per condition). These parameters were used to calculate the total cell numbers in palisade tissue in Figure 6c.
